# Wenlin procedure for treatment of compound thoracic deformity after cardiac surgery

**DOI:** 10.1093/jscr/rjac570

**Published:** 2022-12-09

**Authors:** Wenlin Wang, Weiguang Long, Yang Liu, Bin Cai, Juan Luo

**Affiliations:** Department of Chest Wall Surgery, Guangdong Second Provincial General Hospital, Guangzhou 510317, China; Department of Chest Wall Surgery, Guangdong Second Provincial General Hospital, Guangzhou 510317, China; Department of Chest Wall Surgery, Guangdong Second Provincial General Hospital, Guangzhou 510317, China; Department of Chest Wall Surgery, Guangdong Second Provincial General Hospital, Guangzhou 510317, China; Department of Chest Wall Surgery, Guangdong Second Provincial General Hospital, Guangzhou 510317, China

## Abstract

Cardiac surgery is usually completed through the median sternotomy. If the sternum is not fixed firmly after the operation, it may lead to secondary thoracic deformities. The most common deformity after operation is pectus carinatum, and the relatively less common deformity is pectus excavatum, but the compound thoracic deformity has not been reported. We met a 3-year-old boy who underwent surgery for congenital ventricular septal defect at the age of 1. He developed a severe compound thoracic deformity after surgery. We used Wenlin procedure to correct his deformity and obtained satisfactory results. This article reports the operation of this patient.

## INTRODUCTION

Congenital heart disease is usually treated through median sterntomy [1]. For young children, if the fixation is not firm, secondary thoracic deformities often occurs after surgery. Among these deformities, the most common is secondary pectus carinatum, and secondary pectus excavatum may occasionally occur, but compound deformity has not been reported. The compound deformity has both protrusion and depression on the chest wall. Some authors used the sandwich technique to treat it [2], but some serious cases cannot be treated with this technique. We met a 3-year-old patient with compound thoracic deformity after cardiac surgery. His deformity was quite serious. We performed Wenlin procedure for him and achieved satisfactory results.

## CASE REPORT

**Figure 1:**
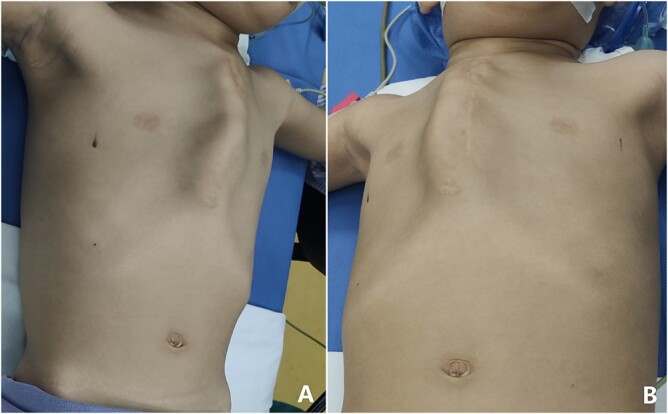
Chest wall appearance before operation. (**A**) Side and (**B**) front view.

**Figure 2:**
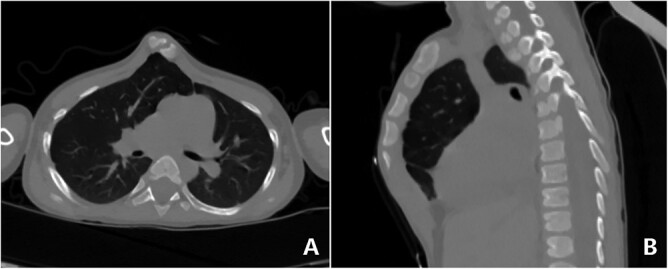
CT examination. (**A**) Section and (**B**) sagittal view.

**Figure 3:**
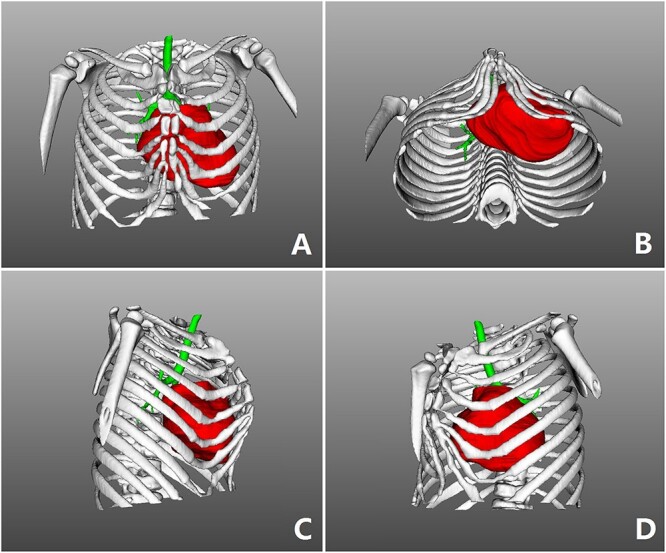
Three-dimensional examination. (**A**) Front view, (**B**) bottom view, (**C**) right side view and (**D**) left side view.

**Figure 4:**
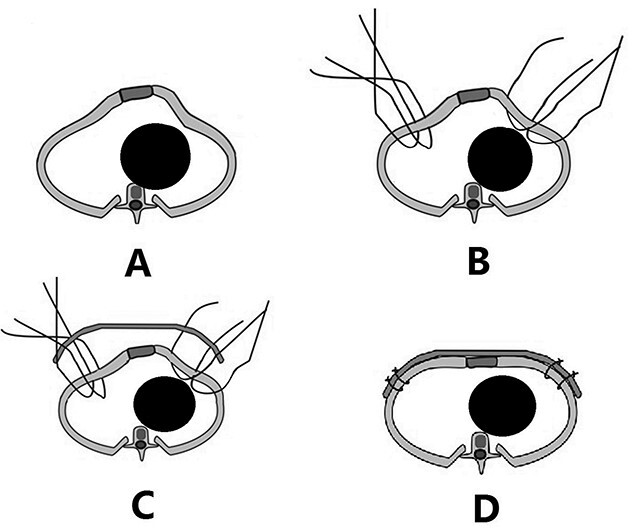
Schematic diagram of Wenlin procedure. (**A**) Section image of deformity; (**B**) position of the fixed steel wires; (**C**) steel bar was placed; and (**D**) after the steel bar is fixed, the median protrusion is flattened and the depressions on both sides are lifted.

**Figure 5:**
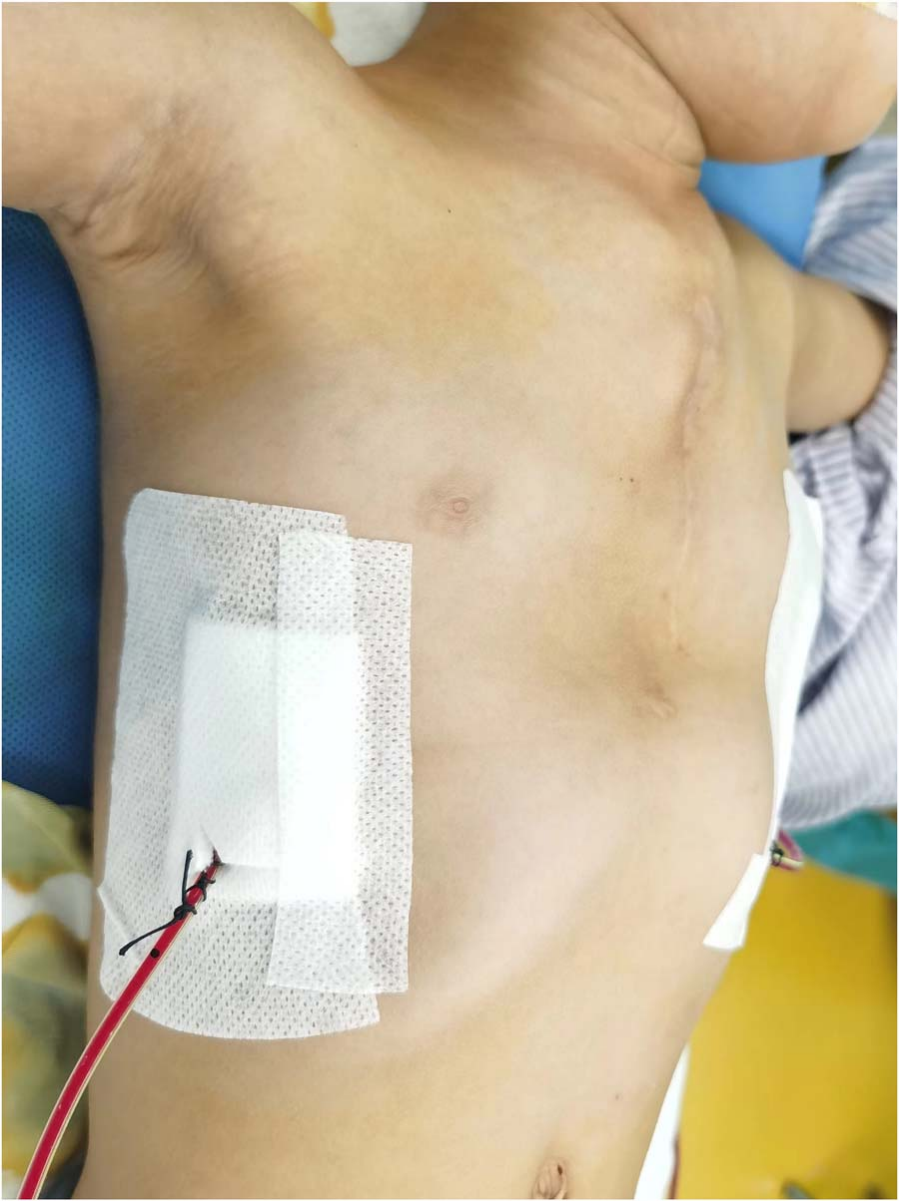
Chest wall appearance after operation.

**Figure 6:**
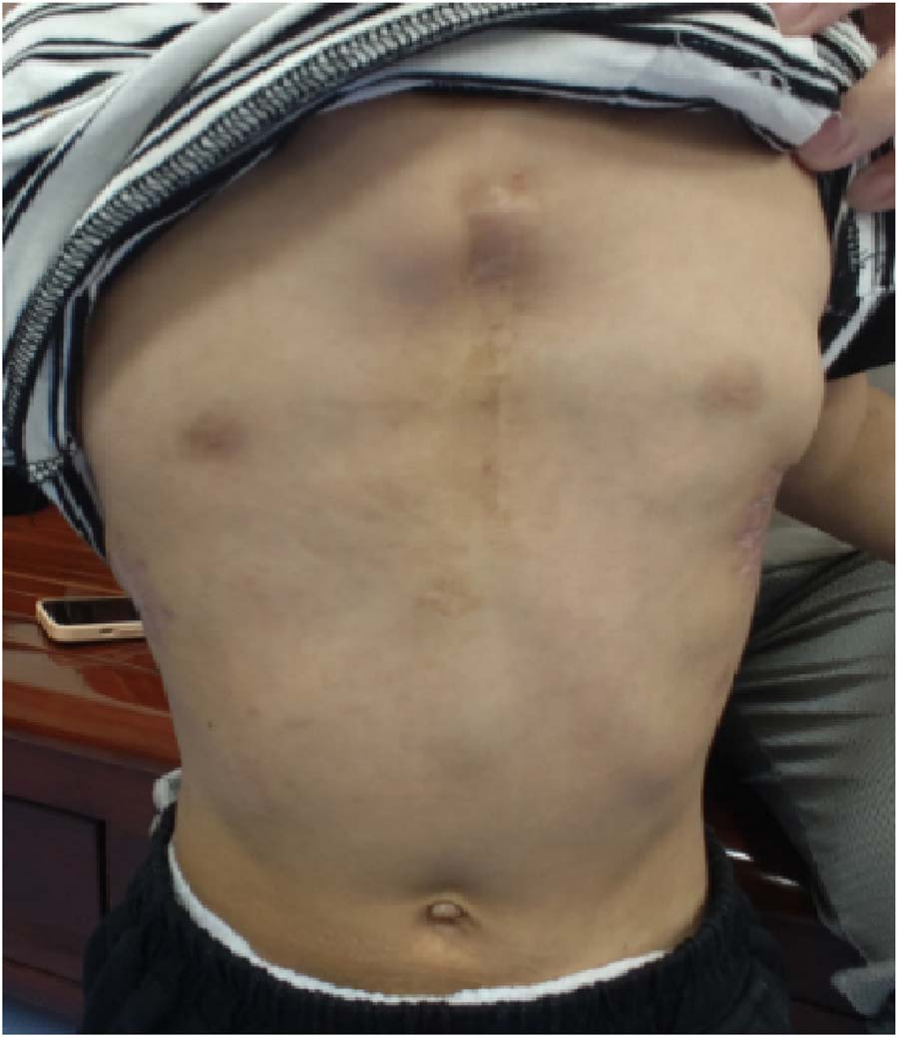
Chest wall appearance 1 year after operation.

The patient was a 3-year-old boy. He was diagnosed with congenital ventricular septal defect shortly after birth, and he underwent heart surgery at the age of 1. The median sternotomy was used in the operation. After operation, the median part of the anterior chest wall is gradually protruded, whereas the bilateral parts of the chest wall are depressed. The deformity worsened with age. In order to correct the deformity, the child was admitted to our hospital at the age of 3. Physical examination showed that there was surgical scar on the anterior chest wall, the median part was protrusive and both lateral parts were obviously depressed (Fig. 1). No heart murmur was heard by auscultation. Imaging examination showed that the anterior chest wall was protrusive in the middle, with acute angle deformity on the top of the protrusion [3], and the lateral chest wall depressed on both sides (Figs 2 and 3). Echocardiography showed that the ventricular septum was integral without residual defect. The patient was diagnosed as secondary severe compound thoracic deformity before operation. After full preparation, the operation was performed under general anesthesia. Two incisions were made on both sides of the chest wall, respectively, with a length of 2 cm. The soft tissues and muscles were dissected to expose the ribs of the side chest wall. Two tunnels were made along the surface of the bone structures, from the incisions to the middle of the chest wall, and connected at the top of the protrusion, with an interval of 3 cm between the tunnels. Two steel bars were put into the tunnels, respectively. After compressing the protrusion with the middle part of the steel bar, two ends of the bar were fixed to two different ribs with steel wires. The interior fixation point was located at the rib of the depression, whereas the outside fixation point was located at the rib of the side chest wall (Fig. 4). After the drainage tube was placed in the operation field, the incision was closed, and the operation was completed. The operation was smooth without complications. The deformity disappeared after the operation (Fig. 5). A follow-up for 1 year showed that the appearance of chest wall was basically normal without obvious deformity (Fig. 6).

## DISCUSSION

The median sternotomy is the standard incision for cardiac surgery [1]. Because the sternum needs to be split through the middle, related complications may occur after the operation. Thoracic bone structures of young children are not fully ossified. When the sternum is split, even after fixation, instability often occurs, which is easy to lead to postoperative thoracic deformities. The most common deformity is the secondary pectus carinatum, which may be related to the impact from the heart beat. Occasionally, pectus excavatum may occur, but its cause is unknown. The postoperative compound thoracic deformity is an extremely rare deformity, which has not been reported previously, and its cause is also unknown. The compound thoracic deformity has both protrusion and depression, but it is not the simple combination of pectus carinatum and pectus excavatum. As the appearance is abnormal and the depression may compress the heart, this deformity needs surgical correction. In the past, the sandwich technique was used to correct common compound thoracic deformities, but in some special cases, the technique cannot achieve satisfactory results [2].

The deformity of this patient is special. His protrusion of the anterior chest wall is extremely serious, which is an acute angle deformity, and there are symmetrical depressions on both sides. Since this kind of deformity cannot be treated by the sandwich technique, we adopted Wenlin procedure for this patient [4, 5]. In the operation, the protrusion was compressed with the middle of the steel bar. As the interior fixation point is located at the rib of the depression, the steel wires can lift the depression and fix it on the steel bar. Therefore, the depression can be eliminated easily. From the perspective of the operating principle, Wenlin procedure is a template plastic surgery [6, 7], so it can complete the correction of various deformities at the same time, including protrusion and depressions.

The highest part of the protrusion of this patient is acute angle deformity, and the correction of this kind of deformity generally requires pre-shaping treatment before the subsequent operation [8]. However, due to the small age of the patient, his bone has a certain elasticity, which makes the direct correction be possible. After compression and fixation with steel bar, the acute angle deformity disappeared completely [3], which laid a foundation for the overall correction of the deformity.

The compound thoracic deformity in this patient is complex and serious, treatment of which is challenging. However, when Wenlin procedure was used, the correction became safe and simple, and satisfactory results were obtained. It can be seen that Wenlin procedure is a useful operation for deformity correction. Even for some compound thoracic deformities, Wenlin procedure can be a reasonable choice.
